# Cross-talk between SIM2s and NFκB regulates cyclooxygenase 2 expression in breast cancer

**DOI:** 10.1186/s13058-019-1224-y

**Published:** 2019-11-29

**Authors:** Garhett L. Wyatt, Lyndsey S. Crump, Chloe M. Young, Veronica M. Wessells, Cole M. McQueen, Steven W. Wall, Tanya L. Gustafson, Yang-Yi Fan, Robert S. Chapkin, Weston W. Porter, Traci R. Lyons

**Affiliations:** 1 Department of Integrative Biosciences, College of Veterinary Medicine, Texas A&M University, College Station, TX USA; 2 Department of Medicine, Division of Medical Oncology, University of Colorado Anschutz Medical Campus, Aurora, USA; 3 The University of Colorado Cancer Center Young Women’s Breast Cancer Translational Program, Aurora, CO USA; 4 Department of Nutrition, Texas A&M University, College Station, TX USA

**Keywords:** SIM2, COX-2, NFκB, Inflammation, Breast cancer, Prostaglandin E_2_

## Abstract

**Background:**

Breast cancer is a leading cause of cancer-related death for women in the USA. Thus, there is an increasing need to investigate novel prognostic markers and therapeutic methods. Inflammation raises challenges in treating and preventing the spread of breast cancer. Specifically, the nuclear factor kappa b (NFκB) pathway contributes to cancer progression by stimulating proliferation and preventing apoptosis. One target gene of this pathway is *PTGS2*, which encodes for cyclooxygenase 2 (COX-2) and is upregulated in 40% of human breast carcinomas. COX-2 is an enzyme involved in the production of prostaglandins, which mediate inflammation. Here, we investigate the effect of Singleminded-2s (SIM2s), a transcriptional tumor suppressor that is implicated in inhibition of tumor growth and metastasis, in regulating NFκB signaling and COX-2.

**Methods:**

For in vitro experiments, reporter luciferase assays were utilized in MCF7 cells to investigate promoter activity of NFκB and SIM2. Real-time PCR, immunoblotting, immunohistochemistry, and chromatin immunoprecipitation assays were performed in SUM159 and MCF7 cells. For in vivo experiments, MCF10DCIS.COM cells stably expressing *SIM2s-FLAG* or *shPTGS2* were injected into SCID mice and subsequent tumors harvested for immunostaining and analysis.

**Results:**

Our results reveal that SIM2 attenuates the activation of NFκB as measured using NFκB-luciferase reporter assay. Furthermore, immunostaining of lysates from breast cancer cells overexpressing SIM2s showed reduction in various NFκB signaling proteins, as well as pAkt, whereas knockdown of SIM2 revealed increases in NFκB signaling proteins and pAkt. Additionally, we show that NFκB signaling can act in a reciprocal manner to decrease expression of *SIM2s*. Likewise, suppressing NFκB translocation in DCIS.COM cells increased *SIM2s* expression*.* We also found that NFκB**/**p65 represses *SIM2* in a dose-dependent manner, and when NFκB is suppressed, the effect on the *SIM2* is negated. Additionally, our ChIP analysis confirms that NFκB/p65 binds directly to *SIM2* promoter site and that the NFκB sites in the SIM2 promoter are required for NFκB-mediated suppression of SIM2s. Finally, overexpression of SIM2s decreases *PTGS2* in vitro, and COX-2 staining in vivo while decreasing *PTGS2* and/or COX-2 activity results in re-expression of SIM2.

**Conclusion:**

Our findings identify a novel role for SIM2s in NFκB signaling and COX-2 expression.

## Background

Ductal carcinoma in situ (DCIS) is a heterogeneous disease characterized by tumor cells that are confined to the ductal system of the breast [1]. DCIS progresses to invasive ductal carcinoma (IDC) through events such as epithelial mesenchymal transition (EMT), basement membrane degradation, inflammatory signaling, and other pathways associated with a wound-healing milieu [2–4]. It is estimated that ~ 20% of mammography-detected breast cancers are DCIS [5] and ~ 65,000 cases of DCIS are diagnosed per year [6]. Provided that DCIS is removed surgically, as is the standard of care, a woman diagnosed with DCIS without recurrence is more likely to die of other causes than of breast cancer [7]. However, it is estimated that ~ 15–20% of DCIS patients develop invasive disease within a decade [8, 9]. Recently identified risk factors for DCIS recurrence include age < 40 at diagnosis, African American ethnicity, hormone receptor negativity, and HER2 positivity [7]. However, these high-risk groups only account for 20% of the DCIS patient population [9]. Therefore, identifying additional risk factors for, or markers that will predict, DCIS aggressiveness is an extremely important goal for preventing invasive cancer in DCIS patients.

There is increasing evidence that inflammation plays a key role in breast cancer progression [10]. One such specific inflammatory pathway is nuclear factor kappa b (NFκB). The NFκB signaling pathway includes five members: NFκB1 (p105/p50), NFκB2 (p100/p52), RelA (p65), RelB, and c-Rel. Dimers of the aforementioned proteins are held in the cytoplasm by inhibitor kappaB kinase (IκB) proteins, primarily IκBα. The mechanism of activation of NFκB requires phosphorylation of IκBα by inhibitor of kappaB kinase (most commonly IKKα and IKKβ), which leads to degradation of IκBα. Upon degradation of the IκBα, NFκB heterodimers, specifically the canonical heterodimer p50/p65, translocate to the nucleus and bind to promoters of target genes, leading to activation of their transcription [11, 12]. Known transcriptional targets of NFκB include mediators of inflammation, such as cytokines/chemokines and immunoreceptors, as well as proteins involved in antigen presentation, cell adhesion, stress response, apoptosis, growth factor receptor signaling, and transcription [13]. Two NFκB consensus sites are located in the promoter region of the human *PTGS2* gene, which encodes for pro-inflammatory enzyme cyclooxygenase 2 (COX-2) [14]. These NFκB consensus sites contribute not only to cancer progression by preventing apoptosis but also to the activation of COX-2-mediated signaling [15]. COX-2 is the inducible form of cyclooxygenase, which is the key enzyme involved in the biosynthesis of the pro-inflammatory prostaglandins [16–21]. Importantly, COX-2 has been implicated in DCIS progression through promotion of proliferation, migration, invasion, and metastatic spread in pre-clinical models [22–24]. Additionally, expression of COX-2 is frequently observed in patients with invasive disease and is associated with DCIS recurrence. Furthermore, the therapeutic benefit of inhibiting COX-2 has been observed in the colon, esophagus, lung, bladder, breast, and prostate cancers [18, 19, 25–35]. Thus, it is logical to expect that inhibition of COX-2 signaling in breast cancer patients could enhance overall prognosis.

Previously, we have shown that Singleminded-2s (SIM2s; expressed from *SIM2*), a member of the bHLH/PAS family of transcription factors, is a tumor suppressor that is expressed in breast epithelial cells and downregulated in the transition from DCIS to IDC [36–39]. Specifically, using the MCF10-DCIS.COM progression model, we demonstrated that re-expression of *SIM2s* inhibits growth, invasive phenotypes, and progression to metastasis. Furthermore, overexpression of SIM2s in breast cancer cells promotes a more luminal-like phenotype, whereas downregulation of *SIM2s* leads to increased invasive potential [39]. Consistent with the role for SIM2s in cancer progression, we have also shown that the NFκB signaling pathway is negatively regulated by SIM2s in normal mammary tissues during postpartum mammary involution [40], which has been identified as a driver of tumor progression and metastasis. In this study, we demonstrate a relationship between SIM2s, the NFκB signaling pathway, and COX-2 in breast cancer cells. We suggest that re-expression of SIM2s could be mediated by inhibition of COX-2 signaling, which may serve to reduce breast cancer progression.

## Methods

### Cell culture

MCF7 and SUM159 cells were purchased from American Type Culture Collection (ATCC) and were maintained in accordance to their guidelines. MCF10A-DCIS.COM (DCIS.COM) cells were generously donated by Dr. Dan Medina (Baylor College of Medicine, Houston, TX, USA). Cells were plated into 6-well plates for RNA isolation experiments according the guidelines from ThermoFisher Scientific. Celecoxib experiments were performed as follows: cells were first dosed with 10 μM celecoxib for 24 h, then media was changed and treatment was performed at 20 μM celecoxib for 24 h, and then cells were harvested for analysis. DHA and PGE2 experiments on cell lines were performed as follows: cells were dosed with 50 μM DHA or 100 μM for 24 h and then harvested for analysis.

### Generation of cell lines

Point mutations in the SIM2 gene were generated via long cDNA synthesis (Invitrogen). Plasmids were amplified using Subcloning Efficiency DH5a competent cells (Life Technologies). Plasmid DNA was isolated using the HiPure Plasmid Maxiprep Kit (Life Technologies) or the ZymoPURE Plasmid DNA Isolation Kit (Zymo Research). Viral transduction was performed as previously described [38]. Puromycin selection (2 μg/mL) was started the following day and maintained for a week.

### Animal models

Two hundred thousand DCIS.COM cells stably expressing anti-COX-2 shRNAs (a generous gift from Kornelia Polyak and Andriy Marusyk) were orthotopically injected and tumors harvested as previously described [22, 23].

### RNA isolation and real-time PCR

RNA isolation and real-time PCR were performed as previously described [39]. Primers can be found in Additional file 1: Table S2.

### Immunoblotting

Cells were washed with cold PBS and lysed in high-salt lysis buffer (50 mM HEPES, 500 mM NaCl, 1.5 mM MgCl_2_, 1 mM ethylenediaminetetraacetic acid (EDTA), 10% glycerol, 1% Triton X-100, pH 7.5) supplemented with 1 mM Na_3_VO_4_ (Sigma) and 1 mM complete ULTRA tablets mini EDTA-free Easy pack (Roche). Protein concentration was determined using the DC Protein Assay (Bio-Rad) with bovine serum albumin as a standard. Immunoblotting and zymography were then conducted as previously described [38]. Antibodies can be found in Additional file 1: Table S1. Blots were imaged on a ChemiDoc MP (Bio-Rad) after incubating in ProSignal Pico ECL Spray (Genesee Scientific) for 3 min. Quantification was performed using ImageJ.

### Immunohistochemistry

Immunohistochemistry (IHC) for COX-2 was performed as previously described [22]. Analysis for positive nuclei was performed as previously described [24]. Antibodies can be found in Additional file 1: Table S1.

### Transient transfection

MCF7 or 293T cells were used for transfections for luciferase activity. One hundred nanograms (0.1 μg) of plasmid containing transcription factor was mixed with up to 1 μg (amount varies per construct) of plasmid containing promoter construct. Three microliters of Genejuice (Novagen) was used per microgram of DNA. DNA and Genejuice were mixed in Opti-MEM media (Invitrogen). Protein was harvested 2 days after transfection, using Reporter Lysis Buffer (Promega). Luciferase activity and total protein were measured as described previously [37]. Luciferase activities were normalized to total protein values and are represented as the means ± SE for three wells per condition.

### Chromatin immunoprecipitation

For chromatin immunoprecipitation (ChIP) assays, 2 μg of plasmid containing repressor and 2 μg of plasmid containing the SIM2 promoter construct were transfected into 293T cells in a 10-cm plate. Chromatin was harvested 2 days after transfection.

### Oncomine analysis

Analysis of SIM2 in primary breast cancer versus metastasis was performed using the Oncomine software (oncomine.org). The TCGA dataset was analyzed for SIM2 with the threshold *p* value set at 0.05 and the threshold fold change set at 2. Additionally, using Oncomine gene expression signatures, we evaluated the breast cancer metastasis concept, setting the odds ratio threshold at 2 and the *p* value at 0.01.

### Statistical analysis

To address scientific rigor, all experiments in cell lines and xenografts were conducted at a minimum in biological triplicates and technical duplicates and were repeated three times. Normal distribution was confirmed before conducting unpaired *t* test. ANOVA analysis was performed using JMP Pro 14 to asses that means are in fact different, and then the post hoc Student’s *t* test was performed. Correlation analysis was performed using Prism7; Pearson’s *r* and a two-tailed test were performed to examine significance. Significance was considered at *p* < 0.05 unless otherwise noted.

## Results

### SIM2s downregulates NFκB signaling

**Fig. 1:**
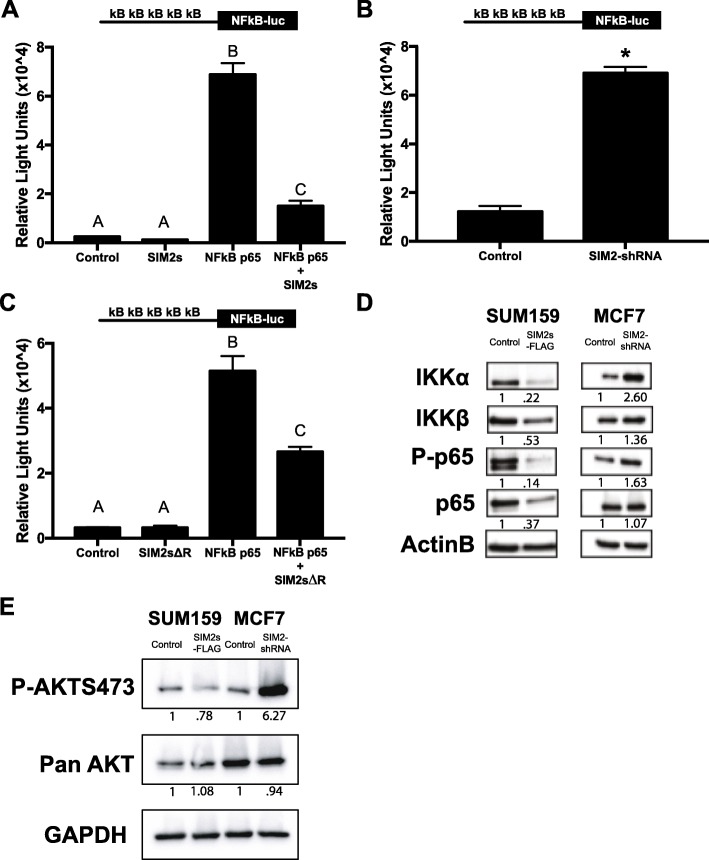
**a** Luciferase activity in MCF7 cells co-transfected with 5x kB binding sites upstream of the luciferase gene (5x NFκB-luc) and NFkB p65 and/or SIM2s. (Diagram of promoter construct is shown above for reference.) **b** Luciferase activity in MCF7 control or MCF7 SIM2-shRNA cells with 5x NFκB-luc. **c** Luciferase activity in MCF7 cells co-transfected with 5x NFκB-luc and NFkB p65 and/or SIM2s with its repression domain deleted (SIM2sΔR). **d** SUM159 plpcx emp (control), SUM159 plpcx SIM2s-FLAG (overexpression), MCF7 psil SCR (control), and MCF7 psil SIM2-shRNA (knockdown) were analyzed by western blot for levels of IKKα, IKKβ, phospho-p65, p65, and beta actin as a loading control. **e** SUM159 plpcx emp (control), SUM159 plpcx SIM2s-FLAG (overexpression), MCF7 psil SCR (control), and MCF7 psil SIM2-shRNA (knockdown) were analyzed by western blot for levels of phospho-AKTs473, pan AKT, and GAPDH as loading control. ANOVA and Student’s *t* test were performed to test significance. **a**, **b**, **c** All significant at *p* < 0.05, *p < 0.05. Analysis was performed via ImageJ for comparison of protein expression

To test the hypothesis that SIM2 negatively regulates NFκB/p65-mediated transcription in breast cancer cells, we co-transfected a reporter plasmid encoding a NFκB binding site upstream of the luciferase gene (5x NFκB-luc) with the p65 subunit along with SIM2s in MCF7 cells and measured relative light units as a readout for NFκB activity. As expected, we observed that p65 strongly activated the reporter construct, but this response was blocked by co-transfection of *SIM2s* (Fig. 1a). Furthermore, we repeated the 5x NFκB-luc transfection in MCF7 cells with stable transduction of a SIM2 sh-RNA or control plasmid (Additional file 1: Figure S1). In the shSIM2 cells, the 5x NFκB-luc was significantly increased over the controls (Fig. 1b). To determine whether inhibition of the 5x NFκB-luc reporter by SIM2s was dependent on the transcriptional repressor activity of SIM2s, the transfection was repeated with a SIM2s expression construct missing the Pro/Ala transcriptional repression domain (SIM2sΔR). Interestingly, this construct also significantly attenuated the activation of the 5x NFκB-luc construct by NFκB/p65, demonstrating that the repression domain of SIM2s is not required for inhibition of NFκB signaling (Fig. 1c; Additional file 1: Figure S2A,B). As an alternative, we performed immunoblot analysis of key players in the NFκB signaling pathway to determine whether SIM2 modulates expression levels of key mediators of NFκB/p65 signaling in our breast cancer cell lines that could downregulate signaling in a posttranscriptional/posttranslational manner. We found that IKKα, IKKβ, phosphorylated-p65, and p65 protein expression, all of which mediate NFκB activation, are decreased in *SIM2s* overexpressing SUM159 cells (Fig. 1d). Similarly, we found that these same NFκB pathway protein levels are increased in *SIM2s* knockdown MCF7 cells (Fig. 1d). These results suggest that SIM2s may affect NFκB-mediated transcription via modulation of protein expression and/or phosphorylation of key mediators of NFκB signaling. Akt is a known activator of NFκB signaling through its ability to phosphorylate and activate IKKα/IKKβ, which leads to nuclear translocation [41, 42]. Thus, we analyzed whether activation/phosphorylation of Akt was modulated by SIM2 expression. Indeed, we observed that overexpression of SIM2s results in a modest decrease in pAkt, while SIM2s knockdown strongly restored pAkt. Together, our results suggest a SIM2s-mediated negative regulation of NFκB/p65 involves de-activation of Akt signaling.

### NFκB signaling downregulates SIM2s expression

**Fig. 2:**
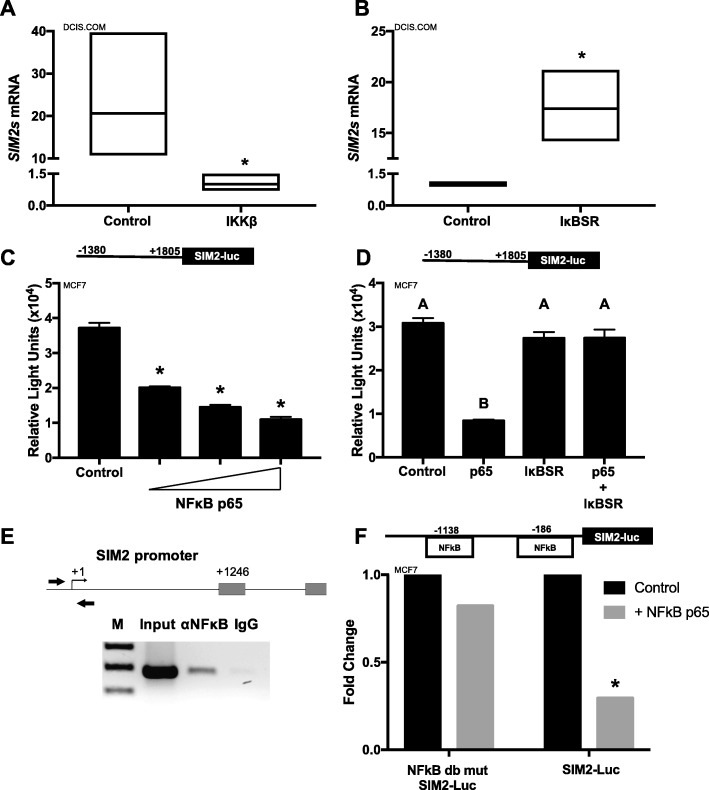
**a** *SIM2s* expression in DCIS.COM control cells and cells overexpressing IKKβ by qPCR as fold change. **b** *SIM2s* expression in DCIS.COM control cells and cells overexpressing IκB-SR by qPCR as fold change. **c** *SIM2*s promoter activity in MCF7 cells co-transfected with SIM2 promoter upstream of the luciferase gene and increasing amounts of NFκB p65 (50 ng, 100 ng, and 150 ng). **d** *SIM2s* promoter activity in MCF7 cells after co-transfection of promoter with control vector (pcDNA3), NFκB p65, and/or IκB-SR. **e** ChIP assay for NFκB p65 binding after transient transfection of SIM2 promoter with NFκB p65 in HEK293T cells. **f** *SIM2s* promoter activity in MCF7 cells co-transfected with *SIM2s* promoter upstream of the luciferase gene and 150 ng NFκB p65 compared with the *SIM2s* promoter activity in MCF7 cells co-transfected with NFκB double mutant *SIM2s* promoter upstream of the luciferase gene. ANOVA and Student’s *t* test were performed to test significance. **a**, **b** All significant at *p* < 0.05, unpaired *t* test: *p < 0.05

Unexpectedly, we also observed that stable overexpression of inhibitor kappa kinase beta (IKKβ), which normally functions to phosphorylate IκBα in the cytoplasm, allowing for activation of NFκB-mediated signaling, significantly decreases *SIM2s* gene expression in the DCIS.COM cells suggesting a reciprocal relationship between NFκB and SIM2s (Fig. 2a). Conversely, when we suppressed NFκB activation via stable transduction of the inhibitor kappaB alpha super repressor (IκB-SR), which maintains the NFκB heterodimer (p50/p65) in the cytosol, *SIM2s* expression was increased (Fig. 2b). To confirm that activation of NFκB downregulates *SIM2* expression in breast cancer cells, we cloned a 2-kb portion of the *SIM2* promoter upstream of the luciferase gene and co-transfected with increasing amounts of p65 in MCF7 cells. We observed dose-dependent repression of *SIM2s* promoter activity (Fig. 2c; Additional file 1: Figure S2C). Importantly, co-transfection with IκB-SR, as well as IκB-SR with NFκB p65, successfully reversed the downregulation of *SIM2* promoter activity (Fig. 2d; Additional file 1: Figure S2D), suggesting that this response was not a dominant negative effect. Analysis of the *SIM2* promoter identified two consensus NFκB binding sites near the transcriptional start site for *SIM2.* Utilizing ChIP analysis, we showed that p65 directly binds to the *SIM2* promoter around the transcriptional start site (Fig. 2e). To determine whether binding of p65 to the NFκB binding sites is necessary for downregulation of SIM2s expression, we mutated the two NFκB sites in the *SIM2* promoter reporter construct and performed additional co-transfection experiments with p65. The NFκB double mutant site promoter failed to inhibit *SIM2* promoter activity when compared to the wild-type promoter (Fig. 2f; Additional file 1: Figure S2E), implicating a direct interaction of NFκB/p65 on the *SIM2* promoter to repress *SIM2s* transcription. These results suggest that NFκB-mediated transcriptional repression of *SIM2s* may serve to reverse SIM2-mediated downregulation of NFκB signaling, allowing for its activation and promotion of transcription of known pro-inflammatory target genes such as *PTGS2*.

### SIM2s expression downregulates COX2

**Fig. 3:**
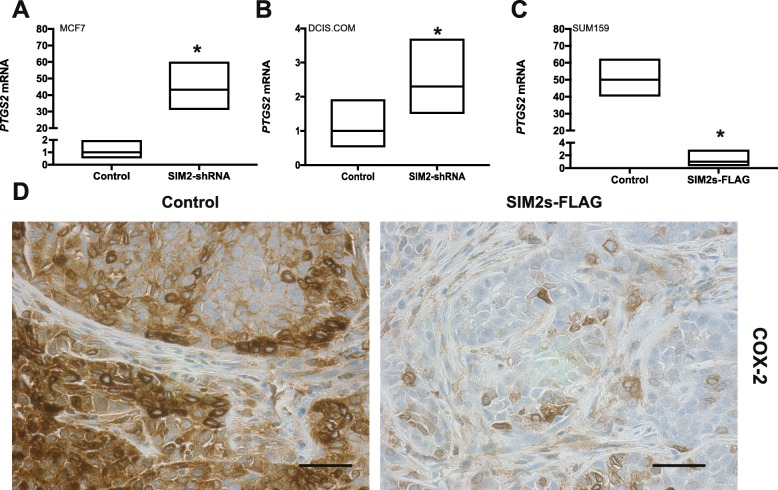
**a** *PTGS2* expression in MCF7 control cells and cells overexpressing SIM2s by real-time qPCR as fold change. **b** *PTGS2* expression in DCIS.COM control cells and cells with SIM2-shRNA by real-time qPCR as fold change. **c** *PTGS2* expression in SUM159 control cells and cells overexpressing SIM2s by real-time qPCR as fold change. **d** Immunohistochemistry for COX-2 in DCIS.COM cells stable transduced with control vector or SIM2s-FLAG (overexpression). Unpaired *t* test: *p < 0.05

To explore the relationship between SIM2s and *PTGS2* expression in breast cancer, we analyzed three different breast cancer cell lines including MCF7, DCIS.COM, and SUM159 cells. The non-invasive MCF7 cell line and the highly invasive triple-negative SUM159 cell line were utilized to examine the differential expression of SIM2s, and subsequent regulation of *PTGS2*, as it relates to invasion. DCIS.COM cells (also triple negative) were used for their unique ability to mimic basal-like DCIS in vivo and their ability to progress to invasive disease upon acquisition of COX-2 protein expression [22, 43]*.* We have previously shown that the invasive competent DCIS.COM cells have more *SIM2s* expression when compared with the non-invasive MCF7 [37, 38]. Confirming and extending this observation, qPCR analysis reveals the lowest *PTGS2* expression in MCF7 cells, which was increased 130-fold in DCIS.COM cells and the highest in the SUM159 cells (Additional file 1: Figure S3). To determine whether reduction of *SIM2s* in the non-invasive cells could increase expression of *PTGS2*, we analyzed control and *shRNA-SIM2s* DCIS.COM and MCF7 cells by qPCR. Our results revealed that downregulation of *SIM2s* led to a significant increase in *PTGS2* gene expression in both cell lines (Fig. 3a, b). Moreover, we found that overexpression of *SIM2s* in highly invasive SUM159 cells significantly inhibited *PTGS2* expression (Fig. 3c). In previous studies, we showed that overexpression of *SIM2s* in DCIS.COM cells blocked invasion in vivo, whereas loss of *SIM2s* or overexpression of the protein product of *PTGS2*, COX-2, resulted in increased invasion and metastasis [22, 39]. To determine the relationship between SIM2s and COX-2 protein expression in vivo, we performed IHC analysis for COX-2 in tumors derived from control and *SIM2s* DCIS.COM xenografts to reveal that COX-2 levels were decreased with overexpression of *SIM2s* (Fig. 3d). Taken together, our results suggest that SIM2s may repress invasion in the DCIS.COM model by promoting downregulation of COX-2.

### COX-2 downregulation restores SIM2s

**Fig. 4:**
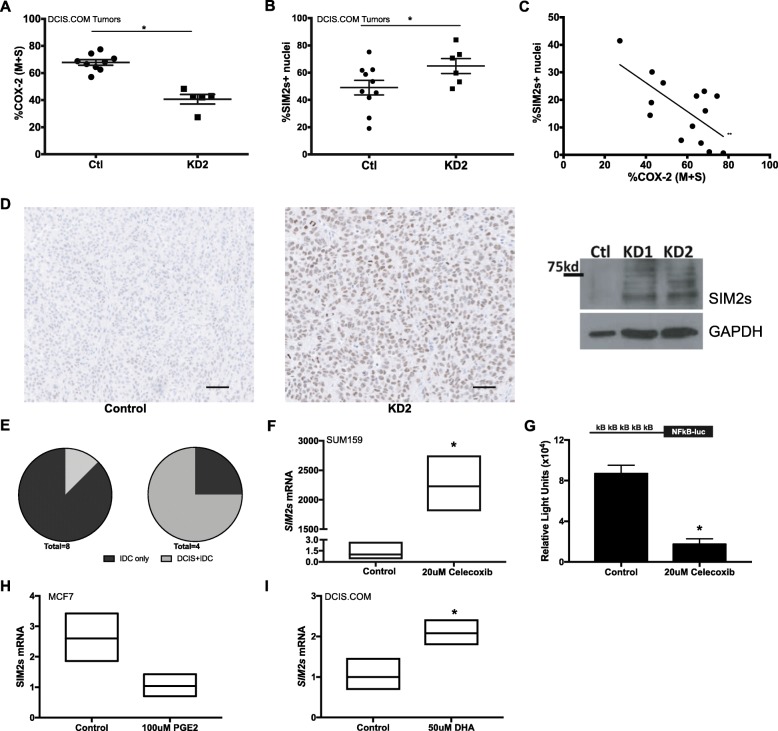
**a** IHC analysis for COX-2 positive nuclei in tumors generated from control (Ctl) and *shPTGS2* (KD2) DCIS.COM cells. Prism7 was utilized for statistical significance. Unpaired *t* test, *p value < 0.02. **b** IHC analysis for SIM2s positive nuclei in tumors generated from control (Ctl) and *shPTGS2* (KD2) DCIS.com cells. Prism7 was utilized for statistical significance. Unpaired *t* test, *p value < 0.0001. **c** Correlation data for SIM2s and COX-2 positive nuclei in tumors generated from control and shPTGS2 DCIS.com cells. Prism7 was utilized for statistical significance. Unpaired *t* test, **p value < 0.01. **d** Images of IHC analysis for SIM2s in tumors generated from control and *shPTGS2* DCIS.COM cells (left); DCIS.COM control, *shPTGS2* (KD1), and *shPTGS2* (KD2) were analyzed by western blot for SIM2s and GAPDH as loading control (right). **e** Pie Chart to show percent tumor progression DCIS+IDC or IDC only in the control group (*n* = 8) and *shPTGS2* (*n* = 4). **f** *SIM2s* expression in SUM159 control cells and cells dosed with 20 μM celecoxib by qPCR as fold change. **g** *SIM2s* expression in DCIS.COM control cells and cells dosed with 50 μM DHA by qPCR as fold change. **h** *SIM2s* expression in MCF7 cells dosed with vehicle or 100 μM PGE2 for 24 h by qPCR, unpaired *t* test: *p* < 0.08. **i** *SIM2s* expression in DCIS.COM cells treated with vehicle (control) or 50 μM DHA by qPCR. Unpaired *t* test: *p < 0.05

Since the invasive potential in DCIS.COM positively correlates with, and depends upon, expression and activity of COX-2 [22, 44], we tested the hypothesis that the loss of invasive phenotype observed with blocking of COX-2 expression was due, in part, to re-expression of *SIM2s*. Thus, we measured SIM2 protein levels by IHC analysis of tumors generated from control and *shPTGS2* DCIS.COM cells, which are less invasive [22]. Our results reveal an increase in positive nuclear staining for SIM2 with *PTGS2* knockdown (Fig. 4a, b; Additional file 1: Figure S4A). We also observed a significant negative correlation between expression of SIM2 and COX-2 and confirmed increased SIM2 in shPTGS2 DCIS.COM and control cells via immunoblot (Fig. 4c, d; Additional file 1: Figure S4B). Additionally, in this study, 87.5% of tumors in the control group had progressed to fully invasive disease compared with 25% in the shPTGS2 group (Fig. 4e). To determine whether COX-2 activity drives the inverse relationship between *SIM2* and *COX-2* and cell invasion, we treated the highly invasive SUM159 cells with a dose of the selective COX-2 inhibitor celecoxib that had previously been shown to decrease invasion of COX-2-expressing cells [22]. We observed a significant increase in SIM2s expression (Fig. 4f). Additionally, celecoxib also inhibited activation of the 5x NFκB-luc reporter MCF7 cells, suggesting that COX-2 activity can also feedback to promote NFκB-mediated suppression of SIM2s (Fig. 4g). Indeed, treatment of MCF7 cells with PGE2, the product of COX-2 activity, inhibited *SIM2s* gene expression (Fig. 4g). Furthermore, we extend our findings to show that docosahexaenoic (DHA), a n-3 polyunsaturated fatty acid (PUFA) that can also result in a shift to a more anti-inflammatory gene expression profile [45] and can reduce COX-2 expression [46–49], significantly increases *SIM2s* expression (Fig. 4i). Thus, our driving hypothesis is that reduction of inflammatory pathways via inhibition of activity and/or decreased COX-2 expression results in re-expression of *SIM2s* and may be one mechanism for preventing progression of DCIS to invasive or metastatic breast cancer [23]. Consistent with this hypothesis, Oncomine analysis reveals that SIM2s is in the top 5–10% of under-expressed genes in a breast cancer metastasis concept signature and in the top 10% of copy number loss genes (Table 1). Further, overall expression is significantly lower in a small number of metastasis in this dataset when compared to the expression in the primaries (Additional file 1: Figure S5), supporting our previous studies showing loss of SIM2s with DCIS progression to IDC [39, 50]. Thus, our results may be relevant for preventing metastasis in women with breast cancer.

**Table 1:** Oncomine analysis

Concept type	Concept name	Size	Dataset	Expression	Top %
Oncomine gene expression signatures	Breast cancer - metastasis - top 5% under-expressed (TCGA breast)	1019	TCGA breast	Under-expressed	5
Oncomine gene expression signatures	Breast cancer - metastasis - top 10% copy number loss (TCGA breast 2)	1881	TCGA breast 2	Copy number loss	10
Oncomine gene expression signatures	Breast cancer - metastasis - top 10% under-expressed (TCGA breast)	2039	TCGA breast	Under-expressed	10

## Discussion

**Fig. 5:**
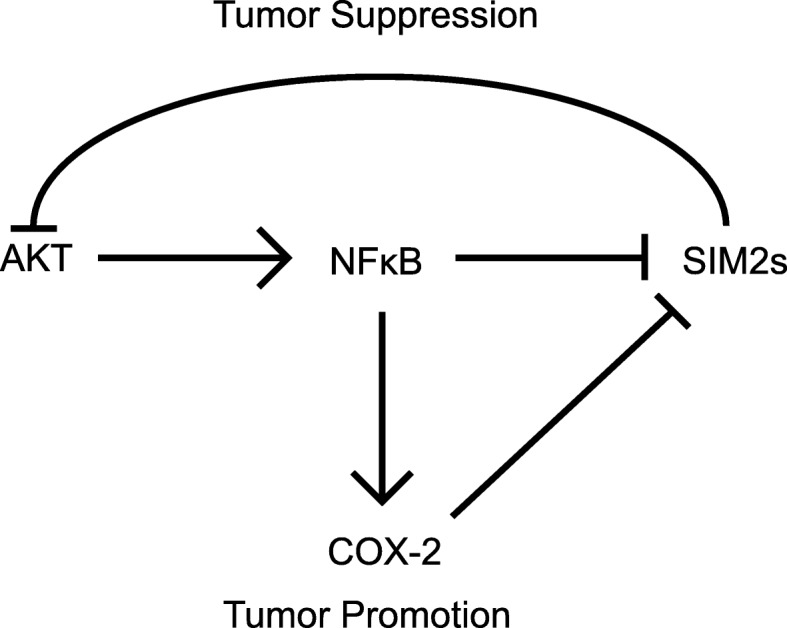
A model depicting SIM2s and NFκB cross-talk regulated to COX-2

Through transgenic mouse models and in vitro studies, SIM2s has been identified as a novel player in several key aspects of mammary gland development. Specifically, genetic ablation of *SIM2s* in mammary epithelial cells revealed that SIM2s is required for ductal morphogenesis and differentiation of luminal cells for milk production during lactation. Furthermore, mammary-specific overexpression of SIM2s resulted in a delay in post-lactational mammary gland involution through suppression of Stat3 and NFκB signaling, as well as maintenance of markers of epithelial cell differentiation normally observed only during lactation. These results suggest that SIM2s has tumor-suppressive activities in the mammary gland through maintenance of epithelial cell differentiation. Consistent with this, loss of *SIM2s* expression in the mammary epithelium results in EMT events, such as loss of E-cadherin and increases in matrix metalloprotease activity, and similar results are also observed in breast cancer cell lines. SIM2s is also downregulated in breast cancer patient samples, further validating its potential role in tumor suppression [39]. In this study, we demonstrate a novel role for SIM2s as a negative regulator of tumorigenesis via downregulation of the NFκB pathway, which normally results in transcriptional activation and expression of the pro-inflammatory/pro-tumorigenic enzyme COX-2, which in turn promotes DCIS invasion. Interestingly, we also identify a novel link between SIM2s and preventing signaling of the pro-tumor/pro-survival kinase Akt, which has been shown to promote tumorigenesis in part through NFκB-mediated COX-2 expression [51]. Additionally, we also show that SIM2s is directly targeted for suppression by NFκB signaling, suggesting a regulatory pro-tumorigenic feedback loop. Consistent with a role for SIM2s preventing this pro-tumorigenic cycle, loss of *SIM2s* also drastically increases COX-2 expression, while loss of COX-2 activity and expression results in re-expression of SIM2s and downregulation of tumor cell invasion. Thus, we have identified a reciprocal relationship between a molecule with known tumor-suppressive activities, SIM2s, and a well-established tumor promotional pathway that involves pro-survival and pro-invasive signaling mediated by Akt, NFκB, and COX-2 (Fig. 5).

Based on our previous results reporting a role for COX-2 in promotion of DCIS invasion [22], and results showing that SIM2 is lost in IDC compared with DCIS in patient samples [39, 50], we predict that loss of SIM2s may be important for progression of in situ lesions to invasive disease via upregulation of COX-2 expression and activity. Consistent with this hypothesis, in the DCIS.COM model, loss of *SIM2s* is associated with increased invasiveness and enhanced tumor aggressiveness and progression, all of which are also observed with gain of COX-2 [22, 23, 37–40, 50]. Specifically, upon loss of SIM2s in tumors, increased co-localization of keratin 5 and vimentin has been observed [39], which is indicative of mesenchymal and invasive phenotypes; furthermore, gain of COX-2 results in increased collagen deposition in the tumor microenvironment, which tumor cells utilize to invade the surrounding tissue and access the vasculature to form metastasis [22, 52, 53]. Matrix metalloproteinases (MMPs), which are associated with basement membrane degradation during mammary gland development and cancer, are also significantly increased with loss of SIM2s [54–56]. These changes likely promote an increased potential for progression of DCIS to IDC and furthermore for tumor cell escape from the primary site. Interestingly, COX-2 has been shown to promote angiogenesis [57] and to inhibit anoikis via activation of Akt [58], suggesting that this pathway can also promote dissemination and survival in circulation. Furthermore, increased COX-2 and increased proliferation are associated with subsequent recurrence of DCIS in patients [21]. Here, we show that cells with low invasive potential exhibit increased expression of COX-2 upon knockdown of *SIM2s* and endogenously express moderate levels of SIM2s compared with the low level of SIM2s observed in the more invasive cells [37]. Likewise, overexpression of SIM2s in invasive cells decreases COX-2 expression. Coincidently, SIM2s overexpression also significantly decreased COX-2 staining in tumor sections and all point toward a role for SIM2 in preventing metastasis. This is consistent with data from the TCGA showing downregulation of SIM2s in a metastasis gene signature. Since it is well known in the literature that COX-2 inhibition is associated with better prognosis for breast cancer patients [59, 60], further studies on strategies for re-expression of SIM2s may be beneficial in improving prognosis of breast cancer patients. Furthermore, an additional implication is that SIM2s could be utilized as a marker to identify DCIS patients that are of low risk for acquisition of COX-2 expression and progression to IDC and/or metastatic disease. However, relevance for this mechanism beyond local invasion, such as in metastatic spread, remains an unanswered question that we will address with future studies.

## Conclusions

These findings support a role for SIM2s in the prevention of breast cancer progression through its ability to repress *PTGS2* expression via modulating the NFκB signaling pathway. It has long been established that NFκB regulates genes involved in cell proliferation and cell survival. Specifically, blocking NFκB in tumor cells can lead to susceptibility to anti-cancer agents. However, due to the complexity of the tumor microenvironment, NFκB signaling also has been found to have anti-cancer effects in various cancer cells. Thus, it is important to investigate a mechanism, specifically in mammary tissue, in which the targeted pathways are highly involved with cell proliferation, survival, migration, and invasion. Due to elevated COX-2 expression correlating with poor prognosis, it is imperative to investigate reducing COX-2/*PTGS2* expression. In the data provided here, we have demonstrated an integral role for SIM2s involvement in mediating NFκB signaling to decrease expression of COX-2/*PTGS2*, which could lead to an improved prognosis for breast cancer patients.

## Supplementary information

**Additional file 1: Figure S1:** Verification of effective transduction knockdown of SIM2 via sh-RNA. Infection of MCF7 cells with the SIM2-shRNA construct results in decreased SIM2 protein levels in comparison to a nonspecific scrambled shRNA construct. **Figure S2:** Verification of NFkB promoter luciferase and SIM2 promoter luciferase assays in the HEK293 cell line. A. Luciferase activity in HEK293T cells co-transfected with 5x kB binding sites upstream of the luciferase gene (5x NFkB-luc) and NFkB p65 and/or SIM2s. (Diagram of promoter construct is shown above for reference.) B. Luciferase activity in HEK293T cells co-transfected with 5x NFkB-luc and NFkB p65 and/or SIM2s with its repression domain deleted (SIM2sΔR). C. SIM2 promoter activity in HEK293T cells co-transfected with SIM2 promoter upstream of the luciferase gene and increasing amounts of NFκB p65 (50ng,100ng, and 150ng). D. SIM2 promoter activity in HEK293T cells after co-transfection of promoter with control vector (pcDNA3), NFκB p65, and/or IκB-SR. E. SIM2 promoter activity in HEK293T cells co-transfected with SIM2 promoter upstream of the luciferase gene and 150ng NFκB p65 compared with the SIM2 promoter activity in HEK293T cells co-transfected with NFκB double mutant SIM2 promoter upstream of the luciferase gene. ANOVA and Student’s t-test was performed to test significance. A, B, C all significant at p<0.05, *p<0.05. **Figure S3:** Verification of *PTGS2* in various breast cancer cell lines. *PTGS2* expression in MCF7, DCIS.COM, and SUM159 parental breast cancer cell lines. ANOVA and Student’s t-test was performed to test significance. A, B, C all significant at p<0.05. **Figure S4:** No correlation between Tumor size and SIM2s or COX-2 gene expression. A. %SIM2s positive nuclei compared to tumor size (cm^2^). B. %COX-2 (M+S) compared to tumor size (cm^2^). Prism7 was utilized for statistical significance analysis. Two-tailed t-test was performed to test significance. **Figure S5:** SIM2 expression in TCGA Breast primary site and metastasis. Oncomine analysis of SIM2 expression in TCGA Breast primary site (n=529) compared to metastasis (n=3). t-Test performed to test significance, p-value=0.006. The Oncomine™ Platform (Thermo Fisher, Ann Arbor, MI) was used for analysis and visualization. For further information, refer to the terms of use. **Table S1:** Antibodies used in study. **Table S2:** Primer sequences used in study.

## Data Availability

Not applicable

## Abbreviations

ChIP: Chromatin immunoprecipitation
COX-2: Cyclooxygenase 2
DCIS: Ductal carcinoma in situ
DHA: Docosahexaenoic acid
EMT: Epithelial mesenchymal transition
IDC: Invasive ductal carcinoma
IKKα: Inhibitor kappa b kinase alpha
IKKβ: Inhibitor kappa b kinase beta
IκB: Inhibitor kappa b
IκBα: Inhibitor kappa b alpha
IκB-SR: Inhibitor kappa b alpha super repressor
NFκB: Nuclear factor kappa b
PUFA: Polyunsaturated fatty acids
SIM2s: Singleminded-2s
