# Supplementary material for: Cross-talk between SIM2s and NFκB regulates cyclooxygenase 2 expression in breast cancer
Source: Breast Cancer Res. 2019 Nov 29;21:131. doi: 10.1186/s13058-019-1224-y (PMC6884910; doi:10.1186/s13058-019-1224-y)
Supplement: Supplementary file 1 — Additional file 1: Figure S1: Verification of effective transduction knockdown of SIM2 via sh-RNA. Infection of MCF7 cells with the SIM2-shRNA construct results in decreased SIM2 protein levels in comparison to a nonspecific scrambled shRNA construct. Figure S2: Verification of NFkB promoter luciferase and SIM2 promoter luciferase assays in the HEK293 cell line. A. Luciferase activity in HEK293T cells co-transfected with 5x kB binding sites upstream of the luciferase gene (5x NFkB-luc) and NFkB p65 and/or SIM2s. (Diagram of promoter construct is shown above for reference.) B. Luciferase activity in HEK293T cells co-transfected with 5x NFkB-luc and NFkB p65 and/or SIM2s with its repression domain deleted (SIM2sΔR). C. SIM2 promoter activity in HEK293T cells co-transfected with SIM2 promoter upstream of the luciferase gene and increasing amounts of NFκB p65 (50ng,100ng, and 150ng). D. SIM2 promoter activity in HEK293T cells after co-transfection of promoter with control vector (pcDNA3), NFκB p65, and/or IκB-SR. E. SIM2 promoter activity in HEK293T cells co-transfected with SIM2 promoter upstream of the luciferase gene and 150ng NFκB p65 compared with the SIM2 promoter activity in HEK293T cells co-transfected with NFκB double mutant SIM2 promoter upstream of the luciferase gene. ANOVA and Student’s t-test was performed to test significance. A, B, C all significant at p<0.05, *p<0.05. Figure S3: Verification of PTGS2 in various breast cancer cell lines. PTGS2 expression in MCF7, DCIS.COM, and SUM159 parental breast cancer cell lines. ANOVA and Student’s t-test was performed to test significance. A, B, C all significant at p<0.05. Figure S4: No correlation between Tumor size and SIM2s or COX-2 gene expression. A. %SIM2s positive nuclei compared to tumor size (cm2). B. %COX-2 (M+S) compared to tumor size (cm2). Prism7 was utilized for statistical significance analysis. Two-tailed t-test was performed to test significance. Figure S5: SIM2 expression in TCGA Breast primary [file 13058_2019_1224_MOESM1_ESM.pdf]

Figure S1

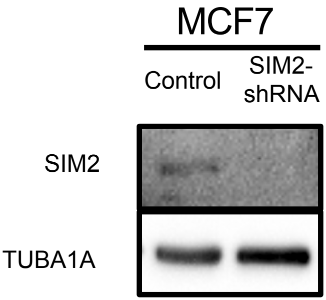

Figure S2

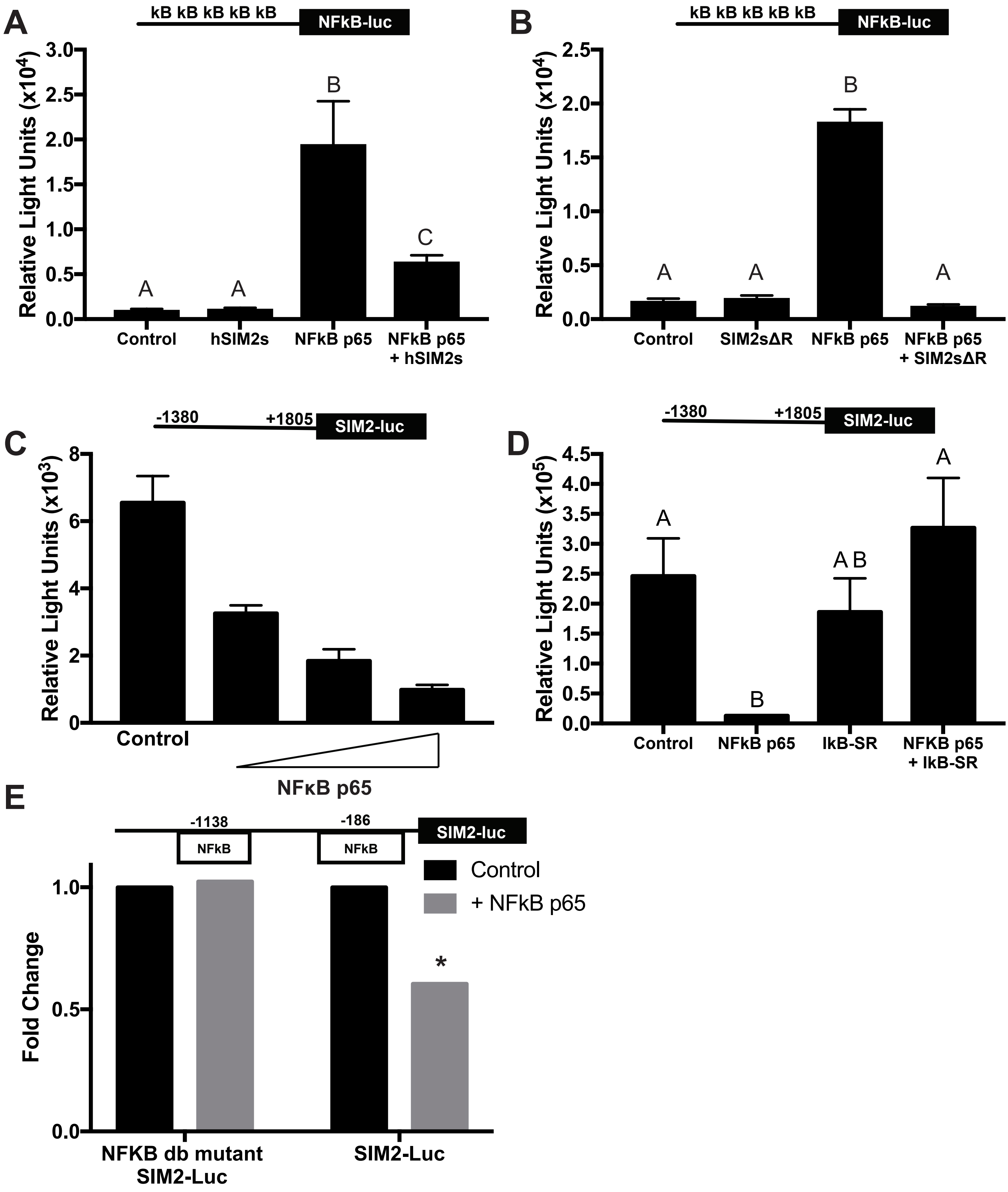

A
kB kB kB kB kB
NFkB-luc
Relative Light Units (x10^4)
3.0
2.5
2.0
1.5
1.0
0.5
0.0
A
A
B
C
Control
hSIM2s
NFkB p65
NFkB p65 + hSIM2s
B
kB kB kB kB kB
NFkB-luc
Relative Light Units (x10^4)
2.5
2.0
1.5
1.0
0.5
0.0
A
A
B
A
Control
SIM2sΔR
NFkB p65
NFkB p65 + SIM2sΔR
C
-1380
+1805
SIM2-luc
Relative Light Units (x10^3)
8
6
4
2
0
Control
NFκB p65
D
-1380
+1805
SIM2-luc
Relative Light Units (x10^5)
4.5
4.0
3.5
3.0
2.5
2.0
1.5
1.0
0.5
0.0
A
B
A B
A
Control
NFkB p65
IkB-SR
NFKB p65 + IkB-SR
E
-1138
-186
NFkB
NFkB
SIM2-luc
Fold Change
1.0
0.5
0.0
Control
+ NFkB p65
*
NFKB db mutant SIM2-Luc
SIM2-Luc

Figure S3

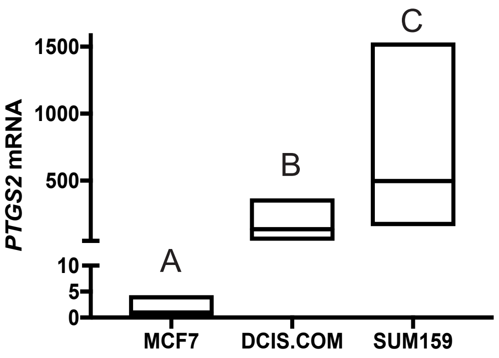

Figure S4

A

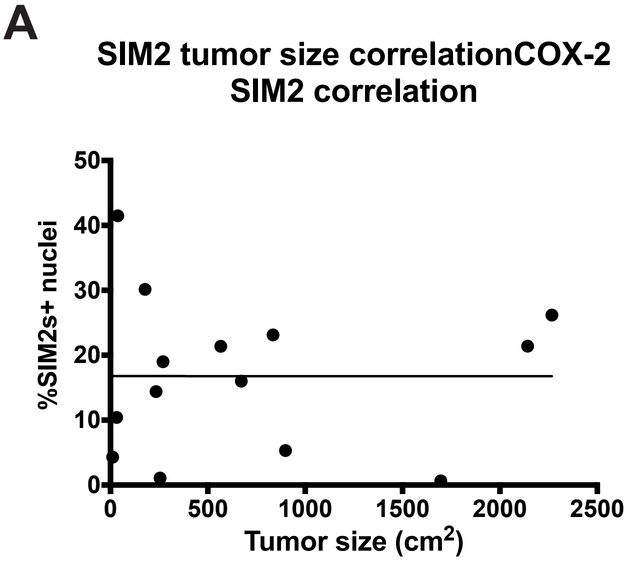

	X vs. Column A
Pearson r	
r	-0.663
95% confidence interval	-0.883 to -0.2043
R squared	0.4396
P value	
P (two-tailed)	0.0098
P value summary	**
Significant? (alpha = 0.05)	Yes
Number of XY Pairs	14

B

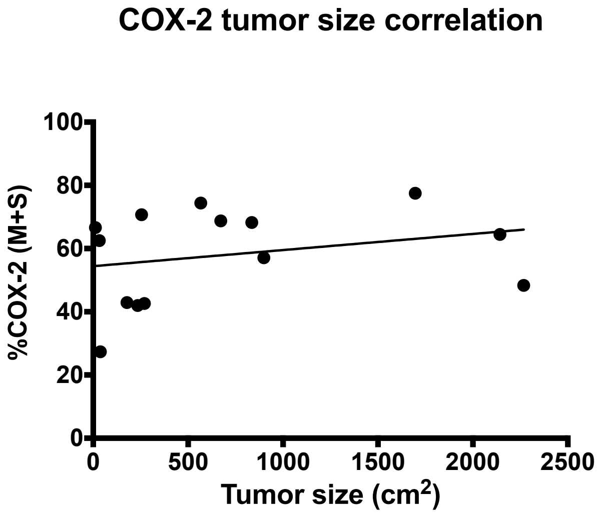

	X vs. Column A
Pearson r	
r	0.2639
95% confidence interval	-0.3101 to 0.6969
R squared	0.06967
P value	
P (two-tailed)	0.3619
P value summary	ns
Significant? (alpha = 0.05)	No
Number of XY Pairs	14

Figure S5

SIM2 Expression in TCGA Breast
Breast Cancer - Metastasis

Under-expression Gene Rank:	837 (in top 5%)	P-value:	0.006
Reporter:	A_23_P211110	t-Test:	-7.522
		Fold Change:	-6.614

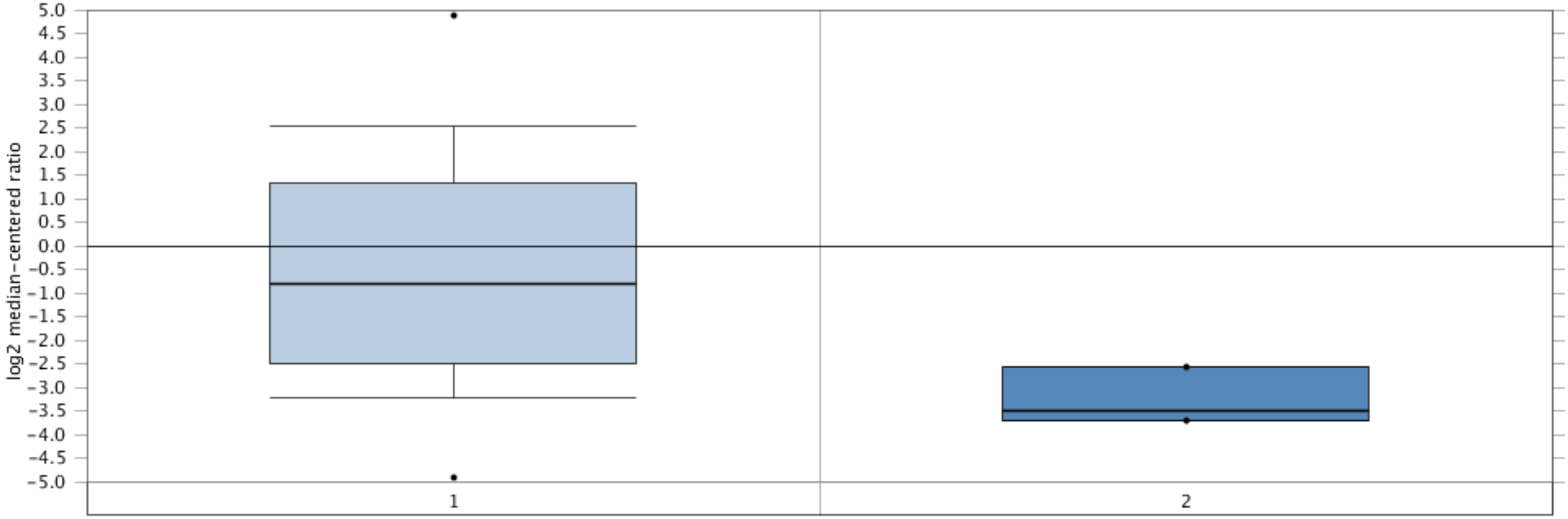

Legend

1. Primary Site (529) 2. Metastasis (3)

TCGA Breast

No Associated Paper 2011/09/02	593 samples
mRNA	20,423 measured genes
Platform not pre-defined in Oncomine	

Table S1

Target	Manufacturer	Product Number	Dilution	Application
SIM2	Millipore	AB4145	1:1000	WB
SIM2	Aviva	Aviva ARP38551_P050	1:500	WB,IHC
ActinB	Cell Signaling Technology	3700s	1:5000	WB
NFkB p65	Cell Signaling Technology	D14E12	1:1000	WB
phospho-NFkB p65	Cell Signaling Technology	93H1	1:1000	WB
IKKβ	Cell Signaling Technology	D30C6	1:1000	WB
IKKα	Cell Signaling Technology	3G12	1:1000	WB
COX-2	Cayman Chemical	160112	1:400	IHC
alpha Tubulin	Abcam	ab4074	1:5000	WB
Akt (pan)	Cell Signaling Technology	4691	1:1000	WB
Phospho-Akt (Ser473)	Cell Signaling Technology	4060	1:1000	WB
GAPDH	Proteintech	60004-1-Ig	1:5000	WB
Anti-Rabbit Secondary	Cell Signaling Technology	7074	1:2000	WB
Anti-Mouse Secondary	Cell Signaling Technology	7073	1:2000	WB

Table S2

	Forward	Reverse
SIM2s	AGGTGGGTCAGGTCTGCTC	GAAGCAGAAAGAGGGCAAGTT
TBP	CGTCCCAGCAGGCAACA	GGTGCAGTTGTGAGAGTCTGTGA
GAPDH	CCAGGTGGTCTCCTCTGACTTC	GTGGTCGTTGAGGGCAATG
18s	CGGCTACCACATCCAAGGAA	CTGGAATTACCGCGGCT
PTGS2	Bio-Rad, PrimePCR	
